# Three-dimensional printed moulds to obtain silicone hearts with congenital defects for paediatric heart-surgeon training

**DOI:** 10.1093/ejcts/ezae079

**Published:** 2024-03-05

**Authors:** Mélanie Frei, Philippe Reymond, Julie Wacker, Mathieu van Steenberghe, Maurice Beghetti, Tornike Sologashvili, Jean-Paul Vallée

**Affiliations:** Radiology Clinics, Diagnostic Department, Geneva University Hospital and University of Geneva, Geneva, Switzerland; Department of Cardiac Surgery, Geneva University Hospital and University of Geneva, Geneva, Switzerland; Charles Hahn Hemodynamic Propulsion Laboratory, Medical Faculty, University of Geneva, Geneva, Switzerland; Department of Women, Children and Adolescents, Paediatric Specialties Service, Geneva University Hospital and University of Geneva, Geneva, Switzerland; Charles Hahn Hemodynamic Propulsion Laboratory, Medical Faculty, University of Geneva, Geneva, Switzerland; Department of Women, Children and Adolescents, Paediatric Specialties Service, Geneva University Hospital and University of Geneva, Geneva, Switzerland; Department of Cardiac Surgery, Geneva University Hospital and University of Geneva, Geneva, Switzerland; Radiology Clinics, Diagnostic Department, Geneva University Hospital and University of Geneva, Geneva, Switzerland

**Keywords:** Surgical simulation, Congenital heart disease, 3D segmentation, 3D modelling, 3D printing, Silicone model

## Abstract

**OBJECTIVES:**

Many types of congenital heart disease are amenable to surgical repair or palliation. The procedures are often challenging and require specific surgical training, with limited real-life exposure and often costly simulation options. Our objective was to create realistic and affordable 3D simulation models of the heart and vessels to improve training.

**METHODS:**

We created moulded vessel models using several materials, to identify the material that best replicated human vascular tissue. This material was then used to make more vessels to train residents in cannulation procedures. Magnetic resonance imaging views of a 23-month-old patient with double-outlet right ventricle were segmented using free open-source software. Re-usable moulds produced by 3D printing served to create a silicone model of the heart, with the same material as the vessels, which was used by a heart surgeon to simulate a Rastelli procedure.

**RESULTS:**

The best material was a soft elastic silicone (Shore A hardness 8). Training on the vessel models decreased the residents’ procedural time and improved their grades on a performance rating scale. The surgeon evaluated the moulded heart model as realistic and was able to perform the Rastelli procedure on it. Even if the valves were poorly represented, it was found to be useful for preintervention training.

**CONCLUSIONS:**

By using free segmentation software, a relatively low-cost silicone and a technique based on re-usable moulds, the cost of obtaining heart models suitable for training in congenital heart defect surgery can be substantially decreased.

**Figure:**
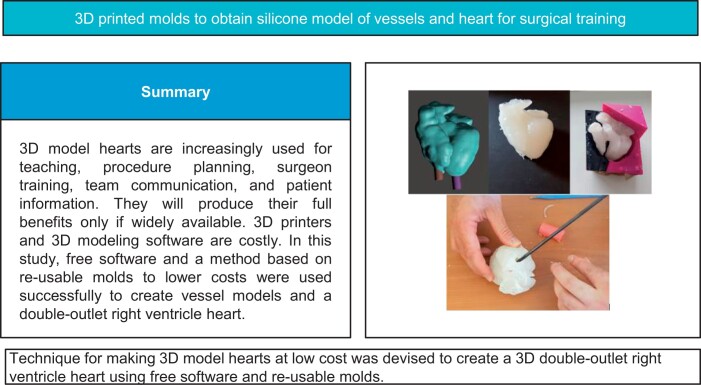

## INTRODUCTION

Surgeons were traditionally trained using the apprenticeship-based model, which involved performing procedures in patients under the supervision of a mentor. A major drawback of this approach is the risk of patient harm due to inexperience. Cadavers have also been widely used but have limited availability and a high cost. The use of live animals is legal in some countries but raises obvious ethical concerns. Neither cadavers nor animals are suitable for training in procedures done to repair or palliate congenital heart disease (CHD), which can be extremely challenging.

The development of new training methods capable of preparing surgeons without exposing patients or animals to potential harm has received considerable interest in recent decades. These methods produce a shift from apprenticeship-based training to a competency-based curriculum, with trainees travelling through successive levels and undergoing standardized assessments at the end of each level. One such method relies on commercially available manikins that replicate the relevant part of the human body. However, manikins are costly and are not available for all surgical procedures. More specifically, no manikins replicating CHDs are available to date.

3D printing can be used to obtain patient-specific models of organs, including hearts with congenital defects [1, 2]. These models can serve to plan surgical procedures [3, 4]. For instance, the use of 3D-printed models has been reported to improve the performance of septal myectomy [5]. The 3DHEART study is currently under way to assess whether 3D-printed models used for planning improve surgical outcomes in babies with CHD. Another use for 3D-printed models is the training of surgical residents [6]. In a study of simulated procedures, 50 experienced surgeons and trainees felt that 3D print CHD models helped to improve surgical skills but that the flexible rubber-like material did not faithfully replicate the consistency and elasticity of the human myocardium [7]. Thus, more work is needed to identify the materials that best imitate the characteristics of human heart tissue.

Decreasing the cost of 3D print model production is another major goal, as lower costs would improve availability. 3D printing requires segmentation of 2D images followed by conversion of the segmented dataset into a 2D representation of the 3D structure, known as the stereolithographic file, which is then fed to the 3D printer. The commercial software packages capable of carrying out this process are expensive. No standard segmentation procedure performed by freeware has been developed to date.

The aim of this study was to create and validate a low-cost and realistic 3D print CHD model suitable for hands-on training in heart surgery. To decrease costs, we used free open-source segmentation software and a method based on re-usable moulds. To obtain a realistic model, we compared several 3D printing materials and silicone types on vessel models, as they were easier to make. The vessels were used by residents to simulate cannulation procedures. A silicone model of a heart with a Taussig-Bing anomaly, double-outlet right ventricle (DORV) with side-by-side great vessels and subpulmonary ventricular septal defect (VSD) was created with the same material and tested by a surgeon.

## METHODS

### Ethics statement

This study has been performed according to the CE 2017-01969 institutional review board approval.

### Production of vessel models and selection of the best material

We created 3D-moulded models of blood vessels using several different polymers to identify the one that best replicated human vascular tissue. We tested all the polymer types available with our 3D printer and then identified the one that produced the softest consistency (Stratasys, Tango Plus FLX 930). We also selected 7 different types of silicone to cover a large range of hardness that could potentially mimic vascular and myocardial tissue (Table 1).

**Table 1:** Main characteristics of the 7 silicones and of the 3D printing material tested in the study

Features	Silbione RTV 4408 A&B	R PRO 10 Reschimica	Bluesil RTV 3410 QC A&B	Wagnersil 17N	Silbione RTV 4420	Wagnersil 22 NF	Wagnersil 25L	Tango Plus FLX 930
Hardness	8	10	12	17	20	22	25	NS
Viscosity (mPa s)	1500	5500	10 000	2400	4000	5000	5500	NS
Elongation at break (%)	650	550	200	320	550	330	500	NS
Completeness of mould filling	+++	+++	+++	+++	+++	+	++	NA
Air bubbles	+	+++	+	–	++	–	+	NA
Cut suture	Very good	Very good	Very good	Very good	Good	Good	Good	Too hard
Cost (US$/kg)	103.0	27	95.0 + 9.0/100 gram catalyzer	35.5	83.7	35.5	32.4	278.2 + 93.9 for support material

**Figure 1:**
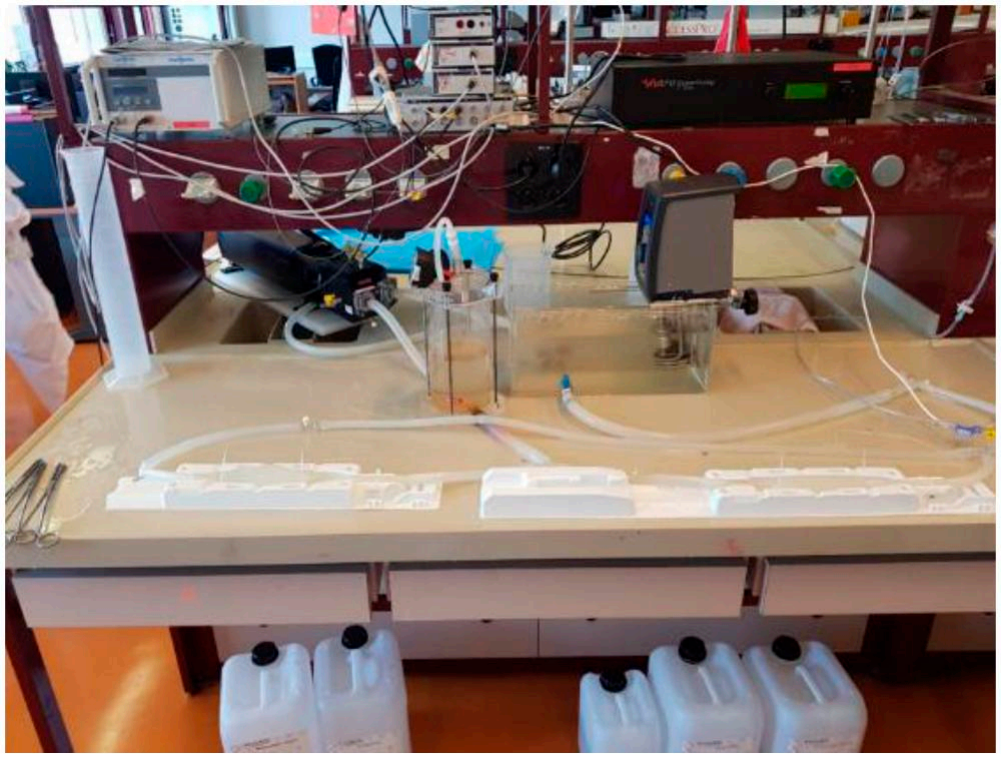
Silicone models of vessels and pump used to circulate blood substitute within them.

**Figure 2:**
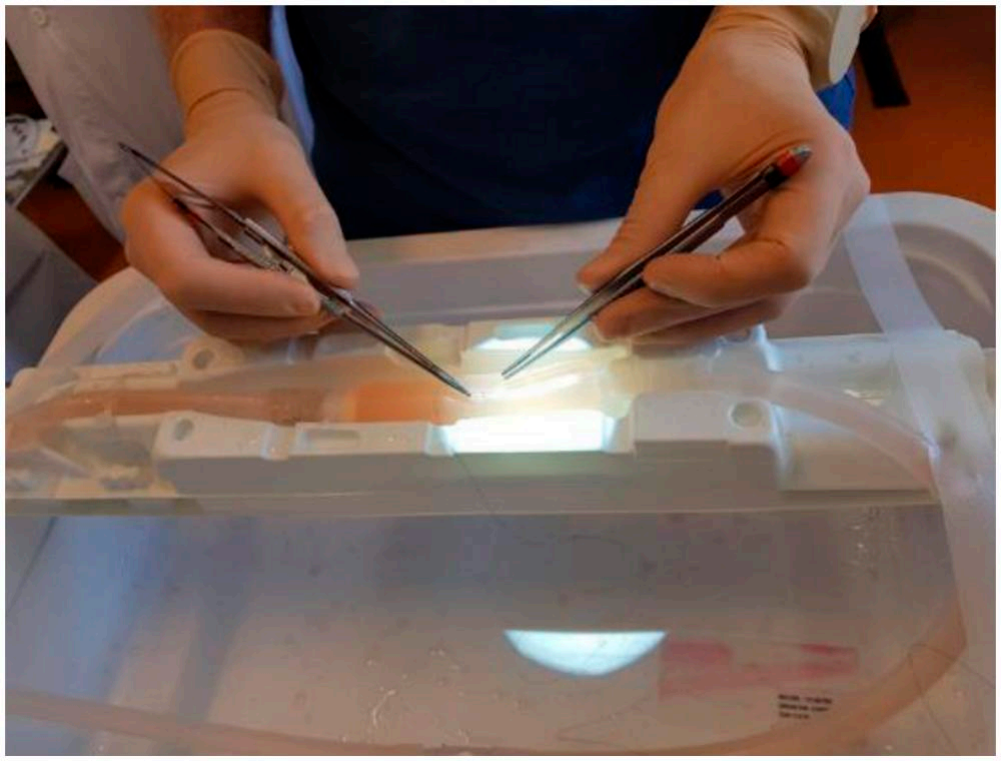
Resident using a 3D vessel model for simulated cannulation.

Tubular moulds for vessels of 3 mm in diameter were 3D printed and then filled using the 7 silicones. One vessel was directly 3D printed with Tango Plus FLX 930 material. For each of these 8 materials, a haemodynamics laboratory researcher (Philippe Reymond) and a medical master’s student (Mélanie Frei) recorded hardness, viscosity and elongation at break based on manufacturer information. They also evaluated the extent of mould filling and the amount of air bubbles within the material through visual inspection. An experienced cardiovascular surgeon (Tornike Sologashvili) cut and sutured each vessel model and gave a subjective assessment of the extent to which each of the 8 materials replicated human vascular tissue on a 3-level Likert scale (‘not good’–‘good’–‘very good’). The material thus identified was used to create additional vessel models, which were connected to a pump and filled with blood substitute circulated at a pressure of 100 mmHg to simulate *in vivo* conditions (Fig. 1). Four surgical residents used these models to practise cannulation, under the supervision of 2 heart surgeons (Fig. 2). They canulated 20 times each and were timed at each canulation and assessed at canulation 1, 10, 15 and 20. The performance of each resident was rated based on procedure time and on the 9 criteria detailed in Supplementary Material Fig. S1.

### Production of the silicone heart model

#### Patient

A magnetic resonance imaging (MRI) scan of the heart obtained as part of standard care in a 23-month-old patient with a DORV was used for this study. The defect was of the Taussig-Bing type, with the subpulmonary VSD and the aorta located anteriorly and to the right of the pulmonary artery. Pulmonary stenosis and right ventricular hypertrophy were also present.

#### Segmentation and 3D printing

We segmented the MRI data using the free open-source software 3D Slicer (http://www.slicer.org) [8]. Segmentation was performed with the ‘grow from seed’ tool then smoothed to obtain a mould with an easy demoulding that could be disassembled without tearing the model. We then used the free open-source software Meshmixer (http://www.meshmixer.com) to prepare the data for the 3D printer Stratasys Objet260 Connex 3 printer (version 29.11.0.19189).

#### Mould making and silicone casting

We created 2 types of moulds, one of the external heart walls and the other of the internal chambers. Wax was poured into the second internal mould. Once hardened, the wax was positioned into the first mould. The best material identified in the vessel-model step (the silicone SILBIONE RTV 4408 A&B, 8 Shore A; Siliconi Italia, Limena, Italy) was poured into the first mould around the wax. The final step consisted in melting the wax to leave only the silicone shell representing the heart walls surrounding the hollow chambers.

**Figure 3:**
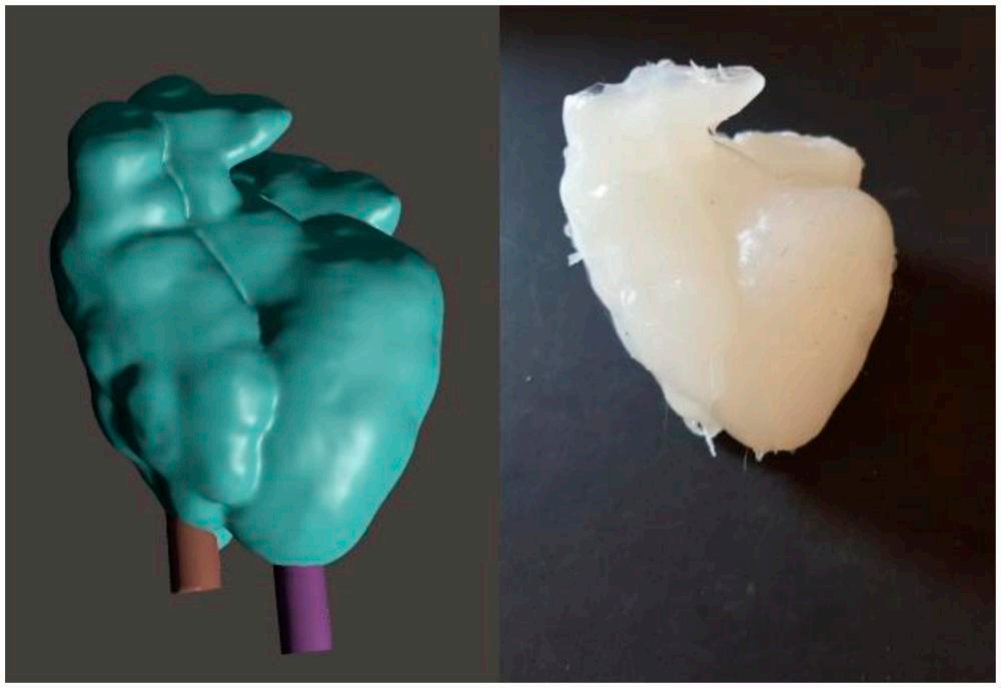
Left: 3D model of the heart created by Meshmixer software, with 2 supports maintaining the wax cast of the chambers in place. Right: silicone model of the heart exhibiting a Taussig-Bing double-outlet right ventricle.

**Figure 4:**
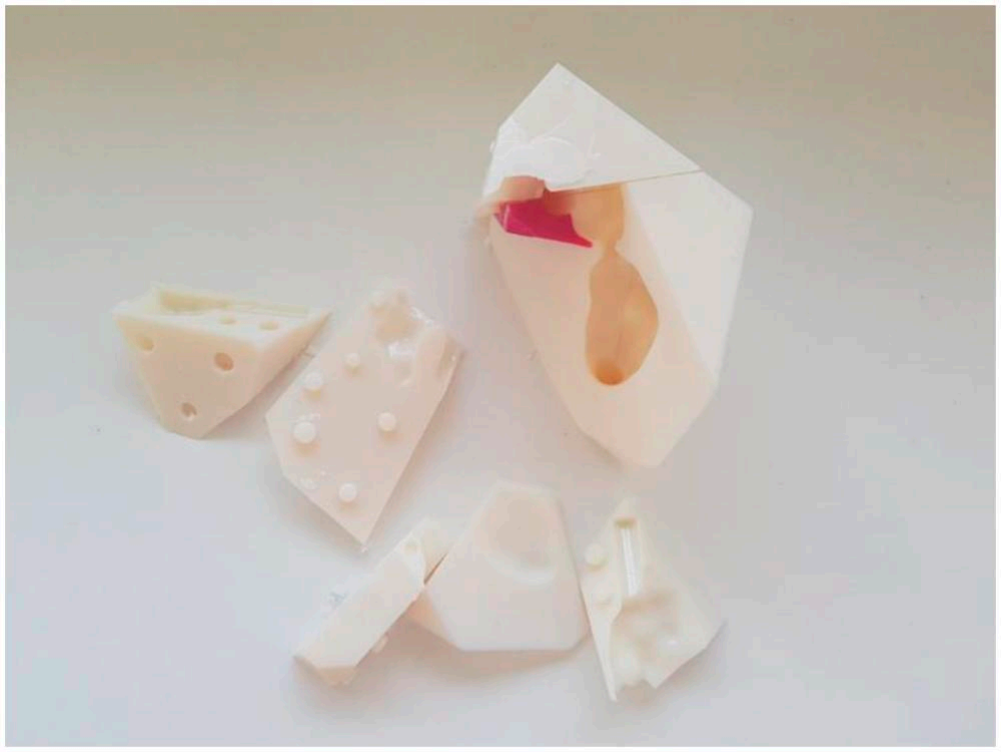
Disassembled wax cast. Note the protuberances and dents that allow reassembling.

For the wax mould, the segmentation dataset for the heart chambers was imported into Meshmixer. To ensure that the hard wax remained stable without touching the wall of the myocardial wall mould, a support was inserted into the apex of each ventricular cavity, using the Meshmixer tool (Fig. 3). A virtual cube was placed around the heart, and Boolean subtraction was performed in Meshmixer to convert it into a mould replicating the shape of the heart cavities. Boolean union was applied to produce a window in the mould through which the melted wax would be poured. Then, the Meshmixer ‘plane cut’ tool was used to cut the wax into multiple small pieces that could be easily unmoulded. Dents and matching protuberances were created to allow connection of the pieces into the chamber-shaped wax cast. Once printed, the mould was greased, assembled and taped to eliminate leaks. Melted wax was then poured in and the mould was refrigerated for several hours (Fig. 4).

**Figure 5:**
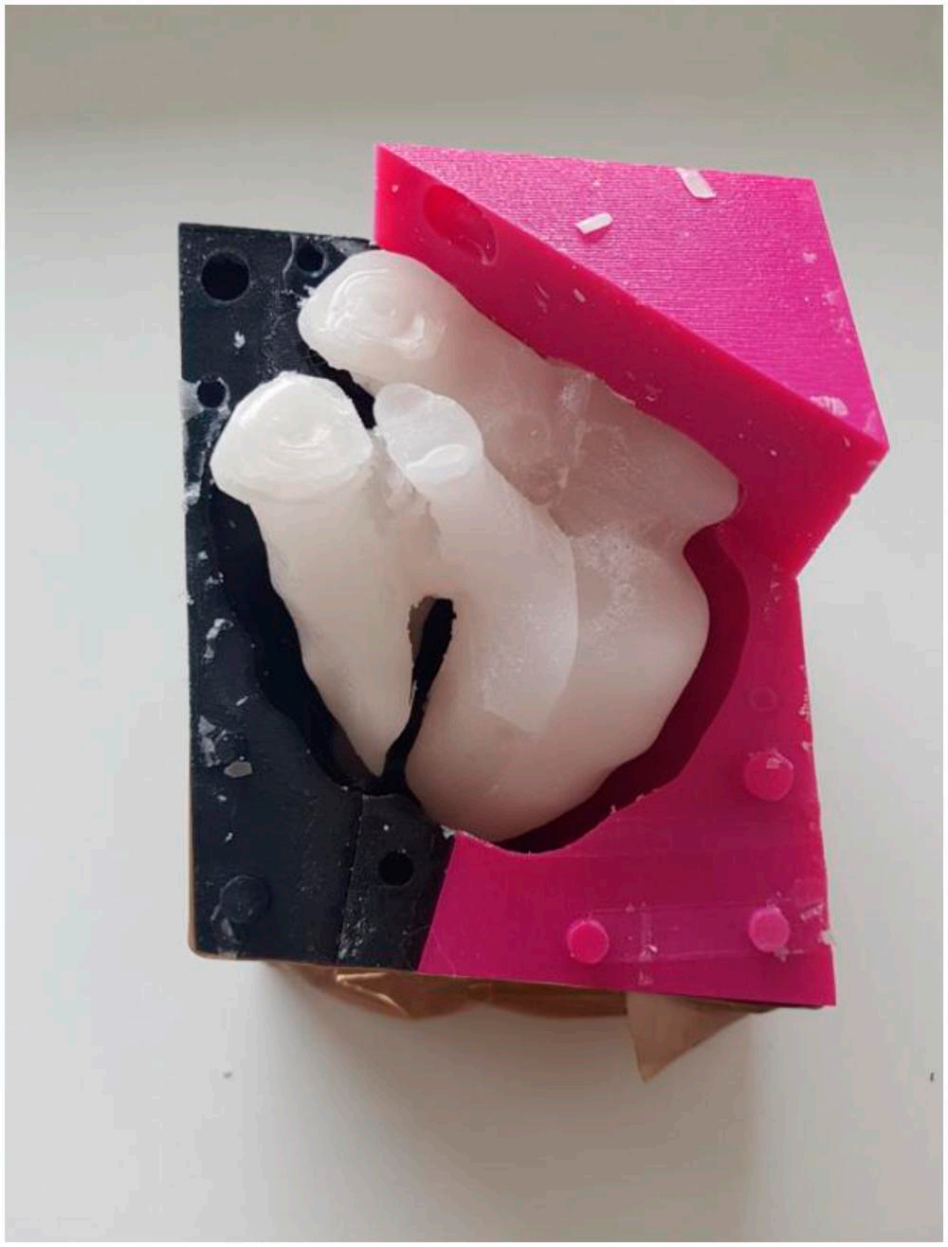
Silicone mould surrounding the hard wax, silicone can be poured between them.

For the mould of the myocardial wall, the segmentation of the whole heart including wall and chambers was imported. Two steps differed from creation of the wax mould. First, a window with the size of a syringe was created for pouring the silicone in at sufficient pressure, thus ensuring complete filling of the mould. Second, a small exit hole was made at the bottom of the mould for extrusion of the air by the silicone. The melting wax was poured inside the chambers’ mould and placed in the fridge for few hours. Once hardened, the wax was placed inside the mould of the myocardial wall (Fig. 5) and the silicone was injected using the syringe between the wax and the mould. The mould was known to be full when silicone reached the exit hole. The silicone required 3 h to solidify. The entire silicone-wax heart was then submerged in hot water to melt the wax, which was removed, leaving only the silicone myocardial wall.

An experiment heart surgeon (Tornike Sologashvili) performed part of a Rastelli procedure on the silicone DORV model. The approach was subaortic, and a silicone patch was implanted to close the VSD. The surgeon evaluated the model and material after the procedure.

### Statistical analysis

The effect of training on procedure time % (procedure time divided by the first procedure time) was estimated by a one-way analysis of variance (ANOVA) with suture time % as an independent variable and the trial number as an effect. When justified by the ANOVA, pairwise comparisons were performed by Tukey’s honestly significant difference *post hoc* test to compare the procedure time % for different trials. The correlation between the procedure time % and the trials and between the performance evaluation and the trials was measured by Pearson's product-moment correlation *r* with the confidence interval in brackets.

## RESULTS

### Selecting the best material

The different materials were assessed by a heart surgeon (Tornike Sologashvili), a haemodynamic laboratory researcher (Philippe Reymond) and a medical master’s student (Mélanie Frei). The tube made of 3D printing material was too hard and therefore ‘not good’. The silicones with 20, 22 and 25 Shore A hardness were assessed as ‘good’ but not sufficiently soft to mimic human tissue. The 4 softer silicones (8, 10, 12 and 17 Shore A) were all rated ‘very good’ based on sufficient softness and flexibility to imitate human vascular and myocardial tissue. Wagnersil 17N silicone (17 Shore A) was not retained due to its blue colour similar to the suture colour. BLUESIL RTV 3410 QC A&B silicone (12 Shore A) was not sufficiently extensible when connected to the pump. R PRO 10 Reschimica (12 Shore A) produced abundant air bubbles, causing leakage of the blood substitute circulating through the tube. The remaining silicone, SILBIONE RTV 4408 A&B (8 Shore A) was the softest, most extensible and easier material and was therefore chosen to create the heart model (Table 1).

### Surgical training

#### Vessel cannulation

The training on the model decreased the procedure time for all the surgeons more rapidly at the beginning and with almost no change after the 15th trial. The suture times were 542 ± 181 s at the first trial, 190 ± 94 s at the 15th trial and 209 ± 72 s at the 20th trial. After the third trial, the procedure time % was significantly smaller than the initial procedure time % (ANOVA <0.0001 and Tukey’s honestly significant difference *post hoc* <0.001 for all these trial numbers). The procedure time % was negatively correlated with the trial numbers (*r* −0.64 [−0.75, −0.49]). The surgeons also improved their technical agility and expertise with the repetition of the suture trial as shown by a positive correlation between the performance evaluation and the trials (*r* 0.78 [0.69, 0.86]). The residents felt that practice on the models helped them acquire the correct gestures and gain confidence.

#### Double-outlet right ventricle

**Figure 6:**
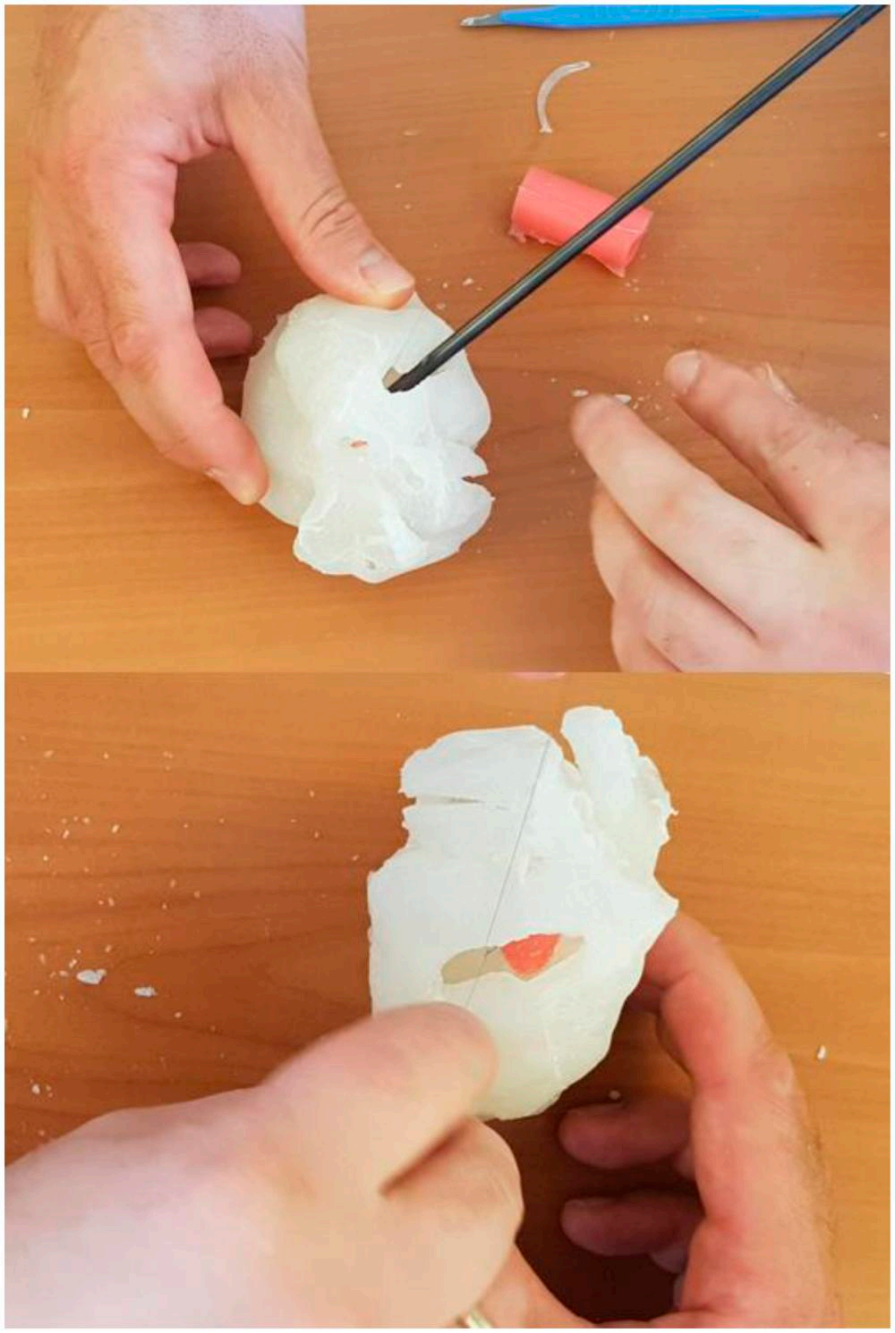
Surgeon using the 3D model of the double-outlet right ventricle heart to simulate the Rastelli procedure, with implantation of a silicone patch.

The heart model faithfully replicated the anatomy of the patient, including the VSD, pulmonary stenosis and right ventricular hypertrophy. An experienced surgeon (Tornike Sologashvili) opened the model via the subaortic approach, explored the inner anatomy and explained the pathology and Rastelli procedure to medical students, residents and surgeons (Fig. 6). Obstructive right ventricular muscle bundles were removed. One of the silicone tubes used to simulate cannulation was cut and used to create a tunnel from the aorta to the left ventricle through the VSD, which was thus closed. The pulmonary valve was not well represented, precluding simulation of pulmonary stenosis repair. However, implantation of a valved conduit would have been possible. The surgeon found that the fragility of the model represented well the fragility of the myocardium. Indeed, too much traction on the suture was tearing the model.

### Cost

With the 3D printing material used in our study, the cost of printing a 200-g heart would have been about 75 US$, without factoring in the cost of the 3D printer. The 3D printing cost of the 2 re-usable moulds, for the wax and silicone, respectively, was about 55 US$, and the cost of the silicone was about 25 US$. SILBIONE RTV 4408 A&B costs about 3 times less than the Tango Plus FLX 930 3D printing material. Thus, given that the moulds were re-usable, the total cost of producing 50 hearts for training would have been about 3750 US$ with direct 3D printing and 1100 US$ with our mould-based silicone method. Additional savings came from the use of free software as opposed to a purchased software suite. Both methods require the use of 3D printer, the price of which varies greatly. We used the hospital’s Stratasys Objet260 Connex 3 printer, which cost around 140 000 US$. More affordable printers such as the Creality Ender-3 V2 FDM 3D Printer allow printing hard objects, such as moulds, in the necessary dimensions, and cost 200 US$.

## DISCUSSION

We created a model of a DORV heart using MRI data from a patient and free open-source software to decrease costs. Further cost decreases were achieved by applying a mould-based technique that produced re-usable moulds of the heart chambers and myocardial wall. We also identified a material, the silicone SILBIONE RTV 4408 A&B, which more closely mimicked human heart tissue than did 6 other silicone types and the most flexible 3D print material available with our printer. We used this silicone to produce the DORV heart model and models of vessels. The latter proved to be effective when used by residents to acquire vessel cannulation skills. The heart model faithfully replicated the patient’s anatomy and was evaluated by an experienced surgeon as constituting a good imitation of human heart tissue.

Several studies have reported the use of 3D heart models for planning intracardiac surgery and for training future heart surgeons [1–7, 9, 10]. In a study of mitral valve modelling, the silicone that best replicated human leaflets out of 7 tested types was Ecoflex 00-30 (Smooth-On, Macungie, PA) [11]. Compared to the silicone found optimal in our tests, Ecoflex 00-30 is softer and more stretchable, but twice as viscous, hypothetically making complete mould filling difficult to achieve. We did not include it in our study.

Valve models obtained directly by 3D printing have been compared to moulded silicone valve models produced using 3D-printed moulds [12]. The 3D printing material was Tango Plus Flex 930 (Stratasys), which has 26–28 Shore A hardness, and the silicone was Dragon Skin and Medium silicone combined with Slacker (Smooth-On). The silicone valves were found to be more realistic than the valves made of 3D printing material. Moreover, successful suture tightening was obtained more often with the silicone than with the 3D print valves. Our work extends the use of the mould approach from valve modelling to the modelling of vessels and hearts. In contrast to valve moulding, 1 major difficulty in the extension of this approach to whole heart moulding is to unmould the 3D model. We solved this problem by dividing the model into multiple small moulds that were fixed together and by using an internal wax mould. The wax mould was unmoulded by heating.

### Limitations

The cost in time and money of making 3D models for surgical training is a major limitation. 3D printers and their consumables are expensive. Many software suites for creating models are also costly. However, we used free software. Moreover, we created moulds that could be re-used to produce several models of the same heart. The material identified as optimal was 3 times less costly than the 3D print material, although it was 3 times more expensive than the Wagnersil materials. We eliminated Wagnersil 17N, rated as providing a very good imitation of human heart tissue, because its blue colour made blue sutures difficult to see. However, other sutures could be used. The total cost of our approach is therefore far lower than an approach provided by 3D printing companies [13].

3D print heart models can be used not only for training surgeons but also for planning procedures, teaching medical students and nurses, enhancing communication among multidisciplinary team members and informing patients and families [14]. These models can also be valuable for assessing the results of surgical procedures, as demonstrated in a 2-year-old with dextro-transposition of the great arteries managed using the Mustard procedure [15]. As a tool for teaching cardiac anatomy to first-year medical students, 3D print models were at least as good as cadavers [16]. In the specific area of CHD, using a 3D print model significantly improved the understanding of criss-cross heart anatomy not only by medical students but also by paediatric cardiology fellows and paediatric cardiologists [17]. 3D print models are particularly useful for planning surgery in patients with complex CHDs, as shown in a 5-month-old with complex aortic arch obstruction managed using the modified Norwood-1 procedure [18] and in 25 patients with complex DORV [19]. A 3D print model of tetralogy of Fallot improved surgical proficiency when used for simulation by 4 residents and 2 fellows [20]. Many other reports in various CHDs and a recent review highlight the benefits of 3D print models in CHD [21]. Clinical studies designed to determine the place of 3D print or moulded models in CHD are now needed [22].

Our moulding method for producing 3D print heart models has several limitations. First, the silicone cast has a single colour. However, multiple colours can be obtained by painting to differentiate components of the heart. Then, valves cannot be fully isolated in MRI or computed tomography datasets. As a result, the pulmonary valve was not well represented in our model. To overcome this issue, the valves could be coloured manually directly on the model. Another option would consist in combining our method with the valve-mould production technique reported by others [11, 23]. Third, our model did not include the coronary arteries. These are well-defined in computed tomography and MRI datasets but adding them would have created difficulties in removing the model from the mould. A separate mould could be made for the coronary arteries, which could then be affixed to the heart using silicone glue. Finally, our DORV model was tried by only 1 surgeon mainly for a proof of concept. Further studies on the benefit of such approach for surgical training remain to be performed.

## CONCLUSION

We developed a low-cost method for creating a heart carrying a complex congenital defect. Free software was used for segmentation to decrease the costs. Moreover, the model production technique was based on moulding, which allowed the use of a silicone that was far less expensive than 3D printing material. Finally, moulds can be re-used many times, providing further cost savings. Our heart model was highly realistic. This method may expand the availability of 3D print heart models for teaching, procedure planning, surgical result assessment, surgeon training, communication within teams and patient information.

## Supplementary Material

ezae079_Supplementary_Data

## Data Availability

The data underlying this article will be shared on reasonable request to the corresponding author.

## ABBREVIATIONS

DORV: Double-outlet right ventricle
MRI: Magnetic resonance imaging
VSD: Ventricular septal defect
