# Supplementary material for: Three-dimensional printed moulds to obtain silicone hearts with congenital defects for paediatric heart-surgeon training
Source: Eur J Cardiothorac Surg. 2024 Mar 5;65(3):ezae079. doi: 10.1093/ejcts/ezae079 (PMC10942813; doi:10.1093/ejcts/ezae079)
Supplement: ezae079_Supplementary_Data [file ezae079_supplementary_data.zip › ezae079_Supplementary_Data/ACAT page 1 (1).pdf]

ACAT - Aortic Cannulation Assessment Tool

RESIDENT NAME______________________________YR OF TRAINING____________ DATE_____________
EVALUATOR______________________Repetition Number___________

1. Needle angles 1 2 3 4 5

1	3	5
Not aware of angles<br>Does not consider subsequent angles	Understand angles, not consistent<br>Partial consideration of subsequent angles	Consistent correct angles<br>Consistent adjustment for subsequent angles

Additional Comments:
____
____

2. Bite 1 2 3 4 5

1	3	5
Irregular entry/exit<br>Hesitant, multiple punctures	Mostly regular entry/exit<br>Mostly single puncture	Consistent regular entry/exit<br>Consistent single puncture

Additional Comments:
____
____

3. Spacing 1 2 3 4 5

1	3	5
Uneven/irregular spacing<br>Irregular distance from previous bite	Mostly even spacing<br>Mostly consistent distance from previous bite	Consistent even spacing<br>Consistent distance from previous bite

Additional Comments:
____
____

4. Needle holder use 1 2 3 4 5

1	3	5
Awkward finger placement<br>Unable to rotate instrument<br>Awkward and not facile<br>Inconsistent needle placement	Functional finger placement<br>Hesitant when rotating<br>Moderate facility<br>Generally good placement	Comfortable, smooth finger placement<br>Smooth rotation<br>High facility<br>Consistent proper placement

Additional Comments:
____
____

5. Use of forceps 1 2 3 4 5

1	3	5
Awkward or no traction<br>Unable to expose<br>Not use to stabilize needle	Moderate proper traction<br>Able to assist in exposure<br>Able to stabilize but rough	Consistent proper traction<br>Consistent proper exposure<br>Knows when to stabilize, gentle

Additional Comments:
____
____
