# Supplementary material for: Three-dimensional printed moulds to obtain silicone hearts with congenital defects for paediatric heart-surgeon training
Source: Eur J Cardiothorac Surg. 2024 Mar 5;65(3):ezae079. doi: 10.1093/ejcts/ezae079 (PMC10942813; doi:10.1093/ejcts/ezae079)
Supplement: ezae079_Supplementary_Data [file ezae079_supplementary_data.zip › ezae079_Supplementary_Data/ACAT page 2.pdf]

	1	2	3	4	5
6. Needle transfer	Marked hesitation in mounting needle		Able to mount needle with hand and partial manipulation	Able to mount needle and manipulate needle easily	

Additional Comments:

____

____

	1	2	3	4	5
7. Scalpel control	Not perpendicular<br>Too big or too small aortotomy<br>Significant leakage		Somewhat perpendicular<br>Close to appropriate aortotomy<br>Some leakage		Perpendicular<br>Appropriate size aortotomy<br>No leakage

Additional Comments:

____

____

	1	2	3	4	5
8. Cannula placement	Improper orientation<br>Too deep or shallow		Somewhat improper<br>Readjusted, good position		Proper orientation<br>Perfect position

Additional Comments:

____

____

	1	2	3	4	5
9. Securing cannula	Too loose, too tight<br>Awkward finger/hand motion		Somewhat loose, tight<br>Hesitant finger/hand motion		Appropriately snug<br>Smooth, comfortable motion

Additional Comments:
